# Isolation, characterization, and genomic analysis of a novel bacteriophage vB_Kp_XP4 targeting hypervirulent and multidrug-resistant *Klebsiella pneumoniae*

**DOI:** 10.3389/fmicb.2025.1491961

**Published:** 2025-03-07

**Authors:** Xiaocui Peng, Jianliang Chang, Hongxia Zhang, Xiaoyu Li, Changhong Zhang, Shiyan Jiao, Chengxiu Lv, Na Wang, Jun Zhao, Bu Wang, Wei Zhang, Zhihua Zhang

**Affiliations:** ^1^Department of Postgraduate, Hebei North University, Zhangjiakou, China; ^2^Respiratory and Critical Care Medicine Department, The First Affiliated Hospital of Hebei North University, Zhangjiakou, China; ^3^Department of Clinical Laboratory, Zibo First Hospital, Zibo, China; ^4^Central Laboratory, The First Affiliated Hospital of Hebei North University, Zhangjiakou, China

**Keywords:** phage therapy, biological characteristics, whole-genome sequencing, *Klebsiella pneumoniae*, multidrug resistance, hypervirulence

## Abstract

**Introduction:**

Hypervirulent and multidrug-resistant *Klebsiella pneumoniae* (hvKP and MDR-KP) are significant public health threats. This study aimed to isolate a lytic bacteriophage targeting these high-risk strains, systematically characterize its biological properties, genomic features, and therapeutic efficacy, and establish a foundation for clinical phage therapy and novel antimicrobial development.

**Methods:**

The phage vB_Kp_XP4 was isolated from river water using the double-layer agar plate method with the clinically isolated strain P4 as the host. Morphology was analyzed via transmission electron microscopy (TEM). Host range, pH, and thermal stability were assessed using spot assays and OD_630_ measurements. One-step growth curves determined the latent period and burst size. Whole-genome sequencing and phylogenetic analysis were performed. Therapeutic efficacy and safety were evaluated in a *Galleria mellonella* infection model.

**Results:**

TEM revealed Phage vB_Kp_XP4 as a tailed phage with an icosahedral head and a long, flexible tail. It lysed an hvKP strain (carrying *rmp*, *peg*, *iuc*, *iro* genes) and an MDR-KP strain (resistant to carbapenems, fluoroquinolones, etc.), with an optimal MOI of 0.1 and latent period <10 minutes. Stability was maintained at pH 4–11 and ≤70°C. Whole-genome sequencing revealed a linear double-stranded DNA genome of 44,344 bp with a G+C content of 53.80%. The genome comprised 54 coding sequences and lacked lysogenic, virulence, or antibiotic resistance genes. Phylogenetic analysis positioned phage vB_Kp_XP4 as a novel species within the genus *Drulisvirus*, family *Autographiviridae*. In the *Galleria mellonella* model, vB_Kp_XP4 prolonged survival of P4-infected larvae (*P* < 0.001)

**Conclusion:**

Phage vB_Kp_XP4 exhibits high stability, specificity, potent lytic activity, and no undesirable genes, demonstrating effective in vivo therapeutic efficacy, suggest its potential for clinical applications against *Klebsiella pneumoniae* infections. The presence of multiple halos during plaque formation further enhances its research value. The complete genome sequence has been submitted to GenBank under accession number PP663283.

## 1 Introduction

*Klebsiella pneumoniae* is a ubiquitous opportunistic Gram-negative enterobacterium (Beamud et al., 2023). Recent national and international surveys have identified it as one of the predominant clinical isolates, particularly among immunocompromised individuals. It is associated with community-acquired and hospital-acquired infections, including pneumonia, meningitis, urinary tract infections, bacteremia, and liver abscesses (Lee et al., 2017; Lan et al., 2021; Pu et al., 2023). The incidence and mortality rates have shown a steady increase in recent years. According to the 2023 CHINET (China Antimicrobial Surveillance Network) report, *K. pneumoniae* ranks as the second most common clinical isolate after *Escherichia coli*, with a rising prevalence and multiple drug resistance trend. Globally, without effective measures to curb the spread of resistance, the annual death toll is projected to reach 10 million by 2050 (Antimicrobial Resistance Collaborators, 2022; Programme TUE, 2023; Antimicrobial Resistance Collaborators, 2024). On May 17, 2024, the World Health Organization (WHO) updated its list of critical bacterial pathogens, categorizing carbapenem-resistant Enterobacteriaceae within the Critical group (World Health Organization, 2024a). A global alert was issued on July 31 concerning a highly virulent, multidrug-resistant *K. pneumoniae* strain, highlighting its rapid transmission and broad infection range, which poses a global health crisis (World Health Organization, 2024b). In the search for new effective strategies to combat this growing threat, phage therapy has regained global attention (Wang et al., 2021). Advances in genomic sequencing have enhanced the understanding and utilization of bacteriophages. Bacteriophages can specifically infect, lyse bacteria, and co-evolve with them, demonstrating significant potential in treating bacterial infections (Castledine et al., 2022). Increasingly explored as an alternative to antibiotics, phage therapy has shown success in numerous reported case (Brives and Pourraz, 2020). The demand for phages is rising, leading to the establishment of several phage banks worldwide, such as the G. Eliava Institute of Bacteriophages, Microbiology, and Virology in Tbilisi, Georgia (Kutateladze, 2015); the Ludwik Hirszfeld Institute of Immnology and Experimental Therapy, Wrocław, Poland (Międzybrodzki et al., 2012); The Center for Innovative Phage Applications and Therapeutics (IPATH) (UCSD, 2019); The Félix d’Hérelle Reference Center for Bacterial Viruses (Université Laval, 2024); The Leibniz Institute DSMZ-German Collection of Microorganisms and Cell Cultures (DSMZ, 2024); Queen Astrid Military Hospital in Brussels (Pirnay et al., 2024); The IPTC in Israel (Yerushalmy et al., 2023); Phage Australia (Sacher et al., 2022) and Phage Canada (Hufsky et al., 2023), etc. The aim is to achieve effective treatment outcomes for bacterial infections through phage therapy and phage-antibiotic combination therapies. However, given the biological activity of phages, a comprehensive understanding of their biological properties and genomic characteristics is essential to optimize their role as clinical therapeutic agents against infectious diseases.

This study successfully isolated a novel bacteriophage, vB_Kp_XP4, from a natural water source. This phage demonstrates lytic activity against highly virulent and multidrug-resistant strains. Basic experiments were conducted to analyze its biological characteristics. Whole-genome sequencing and analysis techniques were employed to perform comparative genomic analysis, gene annotation, and functional prediction of the phage’s complete genome sequence. These findings provide a material foundation and theoretical basis for the application of bacteriophages in treating *K. pneumoniae* infections.

## 2 Materials and methods

### 2.1 Origin and identification of *Klebsiella pneumoniae* strains

The *Klebsiella pneumoniae* strains used in this experiment were identified using a fully automated microbial identification and susceptibility testing system (BD, phoenix100). The host bacterium P4, verified in preliminary experiments, possesses several virulence genes, including *rmp*, *peg*, *iuc*, and *iro*. The antibiotic resistance of the remaining *K. pneumoniae* strains was assessed using the disk diffusion method and PCR, with PCR amplification specifically targeting the *KPC* gene. The string test was employed to measure the viscosity of *K. pneumoniae* strains (Shon et al., 2013). Bacteria were inoculated on LB agar plates and incubated at 37°C for 16 h. A single colony was then picked to observe the string formation. A positive result, indicating a hypermucoviscous phenotype, was defined by the formation of a viscous string greater than 5 mm in length. A negative result indicated a non-hypermucoviscous phenotype.

### 2.2 Phage isolation, purification, and amplification

Following the method described by Mohammadi et al. (2023), flowing river water samples were collected from the Han River in Xiangyang, Hubei Province, a tributary of the Yangtze River. The samples were centrifuged at 10,000 rpm for 15 min, and the supernatant was collected. The supernatant was then filtered through a 0.22 μm syringe filter and stored in 50 mL centrifuge tubes at 4°C. The culture was grown to the logarithmic phase using *K. pneumoniae* P4 as the host bacterium. Then, mix 1 mL of filtered solution with the host bacteria and incubate overnight in a shaker at 37°C and 160 rpm. After incubation, the mixture was centrifuged at 10,000 rpm for 15 min, followed by filtration to collect the supernatant. A further step involved mixing 100 μL of this supernatant with 100 μL of the host bacterial culture in the logarithmic phase, incubating at 37°C in a shaker at 220 rpm for 15 min. Subsequently, 5 mL of 50°C 0.7–0.8% LB semi-solid medium was added, mixed thoroughly, and quickly poured onto the surface of an LB solid medium. After solidification, the plates were inverted and incubated overnight at 37°C. The double-layer agar plate method (Hyman and Abedon, 2009) was employed to observe the presence of bacteriophage plaques. Upon verification, individual plaques were picked and subjected to multiple rounds of purification to obtain a pure phage. For amplification, 5 mL of purified phage solution was mixed with 5 mL of the host bacterial culture in the logarithmic phase, and a liquid LB medium was added to reach a total volume of 50 mL. This mixture was incubated at 37°C with shaking for 6–8 h. The suspension was then centrifuged at 10,000 rpm for 15 min, followed by filtration through a 0.22 μm pore-size membrane to obtain the supernatant, resulting in an amplified phage solution. The amplified phage solution was aliquoted and stored at −80°C in glycerol for future use.

### 2.3 Examination of phage by transmission electron microscopy

To examine the phage morphology, 20 μL of the amplified phage suspension was pipetted onto a 200-mesh copper grid and allowed to adsorb naturally for 5–10 min. Excess liquid was removed using a filter paper strip, and the grid was air-dried briefly. A 2% phosphotungstic acid solution (20 μL) was then applied to the grid for negative staining and left for 3–5 min. Afterward, the excess stain was removed with a filter paper strip, and the grid was air-dried under an incandescent lamp. The morphology of the phages was then observed using transmission electron microscopy (HITACHI, HT7700, Japan).

### 2.4 Phage host range and efficiency of plating

Host range analysis was performed using a panel of 21 *K. pneumoniae* strains by spot tests as previously described with slight modifications. Briefly, the purified phage stocks were gradient diluted with LB liquid medium and 2 μL of gradient diluted phage concentrate (10^2^–10^9^ PFU/mL) was added to the tested bacterial lawn and incubated at 37°C for 12 h. The presence of clear plaque on the bacterial lawn indicated that the tested strains were susceptible to the phage (Han et al., 2023). All *K. pneumoniae* isolates that were sensitive to phage vB_Kp_XP4 in the spot test assay (*n* = 2) were selected for the determination of the Efficiency of Plating (EOP), following the method described by Khan Mirzaei and Nilsson (2015). The EOP was calculated as the ratio of plaque-forming units (PFU/mL) on a sensitive strain to PFU/mL on the indicator strain. Each combination of bacterial strain and phage dilution was tested in triplicate, and the results are presented as the mean of three observations.

### 2.5 Optimal multiplicity of infection determination

The bactericidal activity of phage vB_Kp_XP4 was assessed by determining its time-killing curves. Phage solutions (500 μL) were mixed with 500 μL of host bacterial culture in the logarithmic phase at varying multiplicities of infection (MOIs) of 10, 1, 0.1, 0.01, 0.001, and 0.0001. The mixtures were incubated at 37°C with shaking at 220 rpm for 1 h. After incubation, the cultures were centrifuged at 10,000 rpm for 8 min, and the supernatant was filtered. The supernatant was then serially diluted, and the phage titer was determined using the double-layer agar plate method. The MOI with the highest phage titer was considered the optimal MOI for this phage. The experiment was repeated three times to ensure accuracy.

### 2.6 Monitoring changes in phage load and the effect of bacteriophages on bacterial morphology

Following the method described by Mohammadi et al. with slight modifications (Feng et al., 2023), the phage suspension was mixed with the host bacterial culture at the optimal multiplicity of infection (MOI) and incubated at 37°C for 15 min. Following incubation, the mixture was subjected to immediate centrifugation at 10,000 rpm, and the supernatant was discarded. The pellet was washed multiple times with LB liquid medium and then resuspended in LB medium. The suspension was incubated in a shaker at 37°C and 180 rpm. Samples (300–400 μL) were collected at 0, 10, 20, 30, 40, 50, 60, 70, 80, 90, 100, 110, and 120 min. Each sample was immediately centrifuged at 10,000 rpm, and the supernatant was filtered through a 0.22 μm pore-size membrane. The filtrate was serially diluted in an LB liquid medium, and 100 μL of each dilution was mixed with 100 μL of host bacterial culture. The mixtures were incubated for 15 min and then plated using the double-layer agar plate method. The plates were inverted and incubated overnight at 37°C to observe plaque formation and calculate the phage titer. Three parallel experiments were conducted for each time point. Burst size was calculated using the following formula: (titer after burst—titer at T0)/ (added phage—titer at T0). The curve of phage titer changes was constructed based on the phage titer at each time point. Perform Gram staining on the P4 strain both before and after bacteriophage treatment within a 12-h period. Bacterial morphology was observed using an optical microscope at 1,000× magnification.

### 2.7 Phage temperature and pH stability

Phage stability under different temperatures was assessed by placing the phage suspension in metal baths at 4°C, 10°C, 20°C, 30°C, 37°C, 40°C, 50°C, 60°C, 70°C, and 80°C for 60 min. For pH stability, the pH of the solutions was adjusted from 2 to 11 using HCl and NaOH. Four mL of phage solution (approximately 10^8^ PFU/mL) was incubated at each pH level for 60 min. Phage solutions were then added to host bacteria cultures at an MOI of 0.1, while control groups received LB medium. These were placed in a shaker at 220 rpm and 37°C. Bacterial OD630 was measured at 30-min intervals to monitor changes in bacterial growth. Measurements were taken continuously for 3 to 12 h, with each group tested in triplicate.

### 2.8 Phage genome sequencing and characterization

#### 2.8.1 Whole genome sequencing and annotation of phage

The DNBSEQ-T7 platform was used for sequencing. To ensure the reliability of subsequent analyses, the raw sequencing data were filtered and quality-controlled using fastp (Chen et al., 2018). This step included adapter trimming and the removal of low-quality reads and reads with a high proportion of ‘N’, resulting in clean reads. The metaSPAdes (Nurk et al., 2017) software was employed for the *de novo* assembly of the clean reads, testing different kmer lengths to achieve optimal assembly results. The clean reads were then aligned to the assembled genome using BWA software (Li and Durbin, 2009). Open reading frames (ORFs) were identified using the NCBI ORFfinder server, with methionine and alternative start codons as initiation codons. The protein sequences were compared to the NR database using Blastp to identify sequences with high similarity. Functional annotation of the genes was performed using eggNOG-mapper (Cantalapiedra et al., 2021), which provided annotations from databases such as COG, GO, KEGG, CAZy, BiGG, and PFAM. Additionally, the predicted ORFs and coding sequences were cross-validated with the ORFs and coding sequences predicted by PHASTER (Seemann, 2014). An additional round of ORF prediction and functional annotation was conducted using RASTtk (Aziz et al., 2008; Overbeek et al., 2014; Brettin et al., 2015) and BV-BRC (Olson et al., 2023) to enhance confidence in the predicted coding genes. The presence of antibiotic-resistance genes within the phage genome was assessed using the resfinder database (Bortolaia et al., 2020). Homology searches against the VFDB (Liu et al., 2022) database were conducted to evaluate the presence of virulence genes. The presence of tRNA in the phage genome was determined using the tRNAscan-SE SearchServer online database.^1^ The DeepTMHMM online tool^2^ was utilized to screen for proteins with transmembrane domains (Hallgren et al., 2022).

Linear genome comparison and visualization of coding regions were performed utilizing Easyfig (Sullivan et al., 2011) and Mauve software (Darling et al., 2004). The lifestyle of the phage was predicted using the PHACTS program (McNair et al., 2012). Sequence similarity for further bioinformatic studies was determined using BLASTp searches in the NCBI database.^3^

#### 2.8.2 Phylogenetic analysis of phage

The phylogenetic analysis of the phage’s core proteins, such as terminase large subunit and tail fiber protein, was conducted using the BLASTp tool in the NCBI database to check for sequence similarity of the amino acid sequences. Phages with homologous amino acid sequences to these phage proteins were selected. A phylogenetic tree was then generated using MAGE11 software (Tamura et al., 2021). The phylogeny was constructed using the Maximum Likelihood method (Rokas and Charlesworth, 2001) and the JTT matrix-based model (Jones et al., 1992), with 1,000 bootstrap replicates to ensure the robustness of the analysis. Meanwhile, we predicted its protein structure using AlphaFold3 (Chen et al., 2024).

### 2.9 Therapeutic effect of phage vB_Kp_XP4 in the *Galleria mellonella* larvae

*Galleria mellonella* larvae model was used to assess the potential *in vivo* efficacy of phage against *K. pneumoniae*. The methods for larval injection and incubation were carried out with reference (Han et al., 2023; Li et al., 2023). The experimental procedures are as follows: the larvae selected were 25 ± 5 mm in length, 300 ± 50 mg in weight, with high activity and no visible black spots on the surface. All injections were performed using a Hamilton syringe into the left or right hind leg. Larvae were considered dead when they did not respond to touch. *In vivo* experiment, the larvae were divided into 12 groups, with 10 larvae randomly selected per group: (i)10 μL PBS injection, (ii) 10 μL of 10^7^ CFU/mL strain P4, (iii) 10 μL of 10^8^ CFU/mL strain P4 injection, (iv) 10 μL of 10^8^ CFU/mL bacteriophage, (v) injection of 10 μL of PBS in the right leg (symmetrical position) at 0 h, (vi) injection of 10 μL of PBS in the right leg after 1 h; (vii-ix) MOI = 0.1 (P4 = 10^8^ CFU/mL), 1 (P4 = 10^7^ CFU/mL), 10 (P4 = 10^7^ CFU/mL), with 10 μL of the corresponding concentration of strain P4 injected into the left hind leg, while 10 μL of bacteriophage with titers of 10^8^, 10^7^, or 10^8^ PFU/mL was injected into the right hind leg, respectively; for MOI = 0.1, 10 μL of 10^8^ CFU/mL strain P4 was injected into the left hind leg in three groups of larvae, followed by 10 μL of 10^7^ PFU/mL bacteriophage in the right hind leg at 1 h, 2 h, and 4 h intervals. After completing the above procedures, all larvae were incubated at 37°C and monitored for mortality every 2 h for a total of 60 h. Survival curves were generated using GraphPad Prism v.10.1 and the survival rates were analyzed using Kaplan–Meier and log-rank test. Differences with *p* < 0.05 were considered statistically significant.

### 2.10 Statistical methods

Statistical analysis and plotting were performed using GraphPad Prism version 10.1 software. Student’s t-test was used for both intra-group and inter-group comparisons, with the significance level set at *p* ≤ 0.05.

## 3 Results

### 3.1 Origin and identification of *Klebsiella pneumoniae* strains

Phage-sensitive strains were tested for antibiotic susceptibility using the disk diffusion method, including imipenem, meropenem, ertapenem, levofloxacin, ceftazidime, ciprofloxacin, and amikacin. Strains resistant to more than three classes of antibiotics were defined as multi-drug resistant (Table 1). The results of capsule typing for the *Klebsiella pneumoniae* strains, along with the amplification of *KPC* and *NDM* genes and the outcomes of the string test, are presented in Table 2.

**Table 1:** Drug sensitivity and virulence genes of phage target strains.

Antibiotic	Strains^**#**^		Virulence gene	Strains	
	P4	10		P4	10
IPM	24/S	10/R	*iucA 1*	+	-
MEM	25/S	7/R	*iucA 2*	+	ND
EPM	24/S	6/R	*iroB 1*	+	-
LEV	12/R	13/R	*iroB 2*	+	ND
CAZ	16/S	6/R	*prmpA*	+	-
CIP	9/R	8/R	*prmpA2*	+	-
AMK	18/S	18/S	*peg-344*	+	-

IPM: imipenem, MEM: meropenem, EPM: ertapenem, LEV: levofloxacin, CAZ: ceftazidime, CIP: ciprofloxacin, AMK: amikacin, ^**#**^: Antibacterial circle diameter (mm); ND: No detection, “+”: positive, “-”: negative; R: Drug resistance, S: sensitive.

**Table 2:** Detail information of bacteria of host range test.

Strains	Deduced K-Type	Resistant gene		Wire drawing experiment	Sensitivity	^a^EOP (%)
		*KPC*	*NDM*			
P4	K1	−	−	+	+	100
1	K64	+	−	−	−	0
2	K64	+	−	−	−	0
3	K64	+	−	−	−	0
4	K64	+	−	−	−	0
5	K64	+	−	+	−	0
6	K19	+	−	−	−	0
7	K64	+	−	−	−	0
8	K64	+	−	−	−	0
9	K64	+	−	−	−	0
10	K19	+	−	−	+	51 ± 8
11	K64	+	−	+	−	0
12	K149	+	−	−	−	0
13	K19	+	−	−	−	0
14	K64	+	−	−	−	0
15	K19	−	−	−	−	0
16	K57	−	−	+	−	0
17	K125	−	−	−	−	0
18	K64	+	−	−	−	0
19	K102	−	+	−	−	0
20	K64	+	−	−	−	0

In this Table “**+**” represents positive; “**-**” represents negative. ^a^EOP (efficiency of plating) is calculated in percent as the PFU/mL of the phages on the test strain divided by the PFU/mL obtained on strain P4 multiplied by 100.

**Figure 1:**
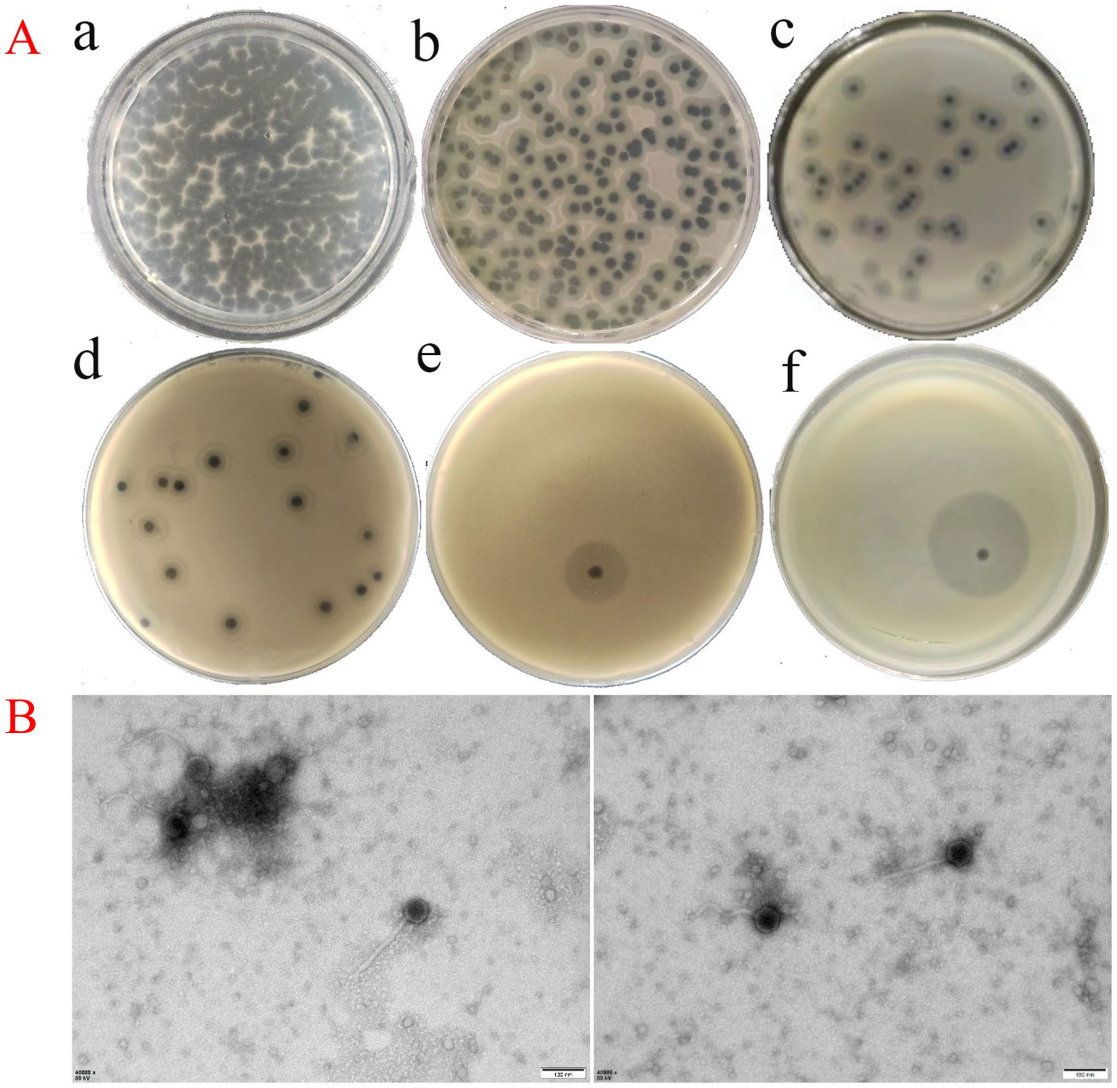
**(A, a–d)** Plaque morphology of phage vB_Kp_XP4 stock solution (≈ 10^9^) at various dilution gradients (10^−3^, 10^−6^, 10^−7^, and 10^−8^), with plaques approximately 2 mm in diameter and surrounded by a halo comprising a multi-layered translucent zone; e: Plaque and halo morphology observed at 24 h post-inoculation; f: Plaque and halo morphology observed at 36 h post-inoculation. **(B)** TEM image of phage vB_Kp_XP4 (scale bar = 100 nm).

Using the hypervirulent *K. pneumoniae* strain P4 as the host, a bacteriophage was isolated and purified from Han River water and named *Klebsiella* phage vB_Kp_XP4. Serial dilutions of the phage stock solution (≈10^9^ PFU/mL) were prepared, and within 12 h of incubation at 36°C on double-layer agar plates, plaques with a diameter of approximately 2 mm formed. These plaques exhibited a halo with multiple semi-transparent layers around them. The plaque morphology at different dilutions (10^−3^, 10^−6^, 10^−7^, 10^−8^) is shown in Figures 1A,a–d. Over time, the halo gradually expanded, with the plaque and halo diameters reaching up to 15 mm at 24 h under ambient conditions (Figures 1A,e) and 28 mm at 36 h (Figures 1A,f).

### 3.2 Examination of phage by transmission electron microscopy

Transmission electron microscopy (TEM) revealed the morphological characteristics of phage vB_Kp_XP4. The phage displayed a typical icosahedral head structure with a diameter of approximately 55 ± 2 nm and a non-contractile tail measuring about 168 ± 10 nm in length (Figure 1B). Based on these morphological features, phage vB_Kp_XP4 was classified as a tailed phage with a flexible, non-contractile tail.

### 3.3 Phage host range and efficiency of plating

The lytic spectrum and efficiency of phage vB_Kp_XP4 were evaluated across 21 *K. pneumoniae* strains. The phage exhibited a lytic rate of 9.5% (2 out of 21 strains). Virulence genes *iucA1*, *iucA2*, *iroB1*, *iroB2*, *prmpA*, *prmpA2*, and *peg-344* were amplified in phage-sensitive strains using PCR. Strains positive for all these genes were defined as ‘high-virulence strains’ (Table 1). These results suggest that phage vB_Kp_XP4 has therapeutic potential against infections caused by hypervirulent or multidrug-resistant *K. pneumoniae*. The EOP analysis showed that when the bacteriophage-to-bacteria ratio is approximately 0.1, phage vB_Kp_XP4 demonstrates extremely high efficiency in lysing the P4 strain, with fewer than 10 colonies growing (EOP ≈ 1). In contrast, the lysis rate for strain 10 is only 0.51 ± 0.08. Detailed information is presented in Table 2.

### 3.4 Optimal multiplicity of infection determination

**Figure 2:**
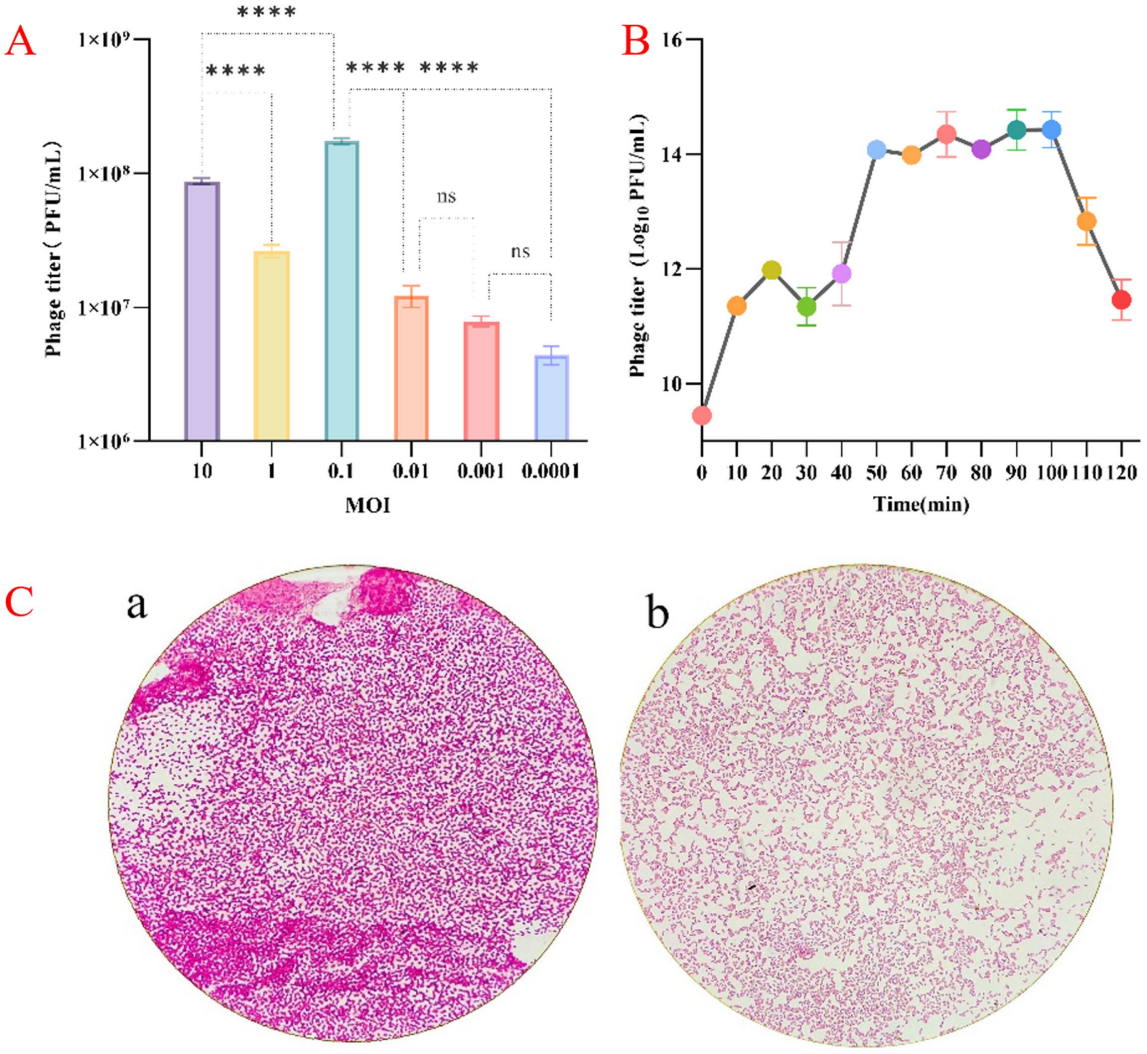
**(A)** Results of the optimal MOI assays for phage vB_Kp_XP4. **(B)** Monitoring changes in phage vB_Kp_XP4 load. **(C, a)** Morphology of the P4 bacterial strain in the absence of phage treatment; **(b)** Morphology of the P4 bacterial strain following exposure to phage vB_Kp_XP4.

*Klebsiella* phage vB_Kp_XP4 was tested for its bactericidal activity against its host, *K. pneumoniae* P4, at various MOIs. At an MOI of 0.1, the phage titer reached approximately 10^8^ PFU/mL, significantly higher than in other groups (Figure 2A).

### 3.5 Monitoring changes in phage load and the effect of bacteriophages on bacterial morphology

The parameters of phage reproduction, including but not limited to the latent period and changes in phage quantity during the growth cycle, are valuable for the practical application of phages. Monitoring changes in phage load (Figure 2B) indicated that phage vB_Kp_XP4 has a relatively short latent period of approximately 10 min. When co-cultured with the host bacteria for 10 to 50 min, the phage titer increased rapidly, and the bacterial suspension transitioned from turbid to clear. After 50 min of co-culture, the phage concentration peaked at 10^14^PFU/mL, entering a plateau phase where the suspension remained relatively clear. The average burst size of this phage was about 387 phages/cell. However, after 100 min, a noticeable decline in phage concentration was observed, and the bacterial suspension gradually became turbid. Under an optical microscope at 1,000× magnification, the morphology of the P4 strain changed after treatment with bacteriophage vB_Kp_XP4. Macroscopically, we observed that the staining intensity in image “a” is higher compared to image “b.” Microscopically, individual bacterial cells appeared smaller after phage treatment than before. This suggests that phages may have disrupted the capsule structure of *Klebsiella pneumoniae*. (Figure 2C). However, this morphological change was not observed in strain 10.

### 3.6 Phage temperature and pH stability

**Figure 3:**
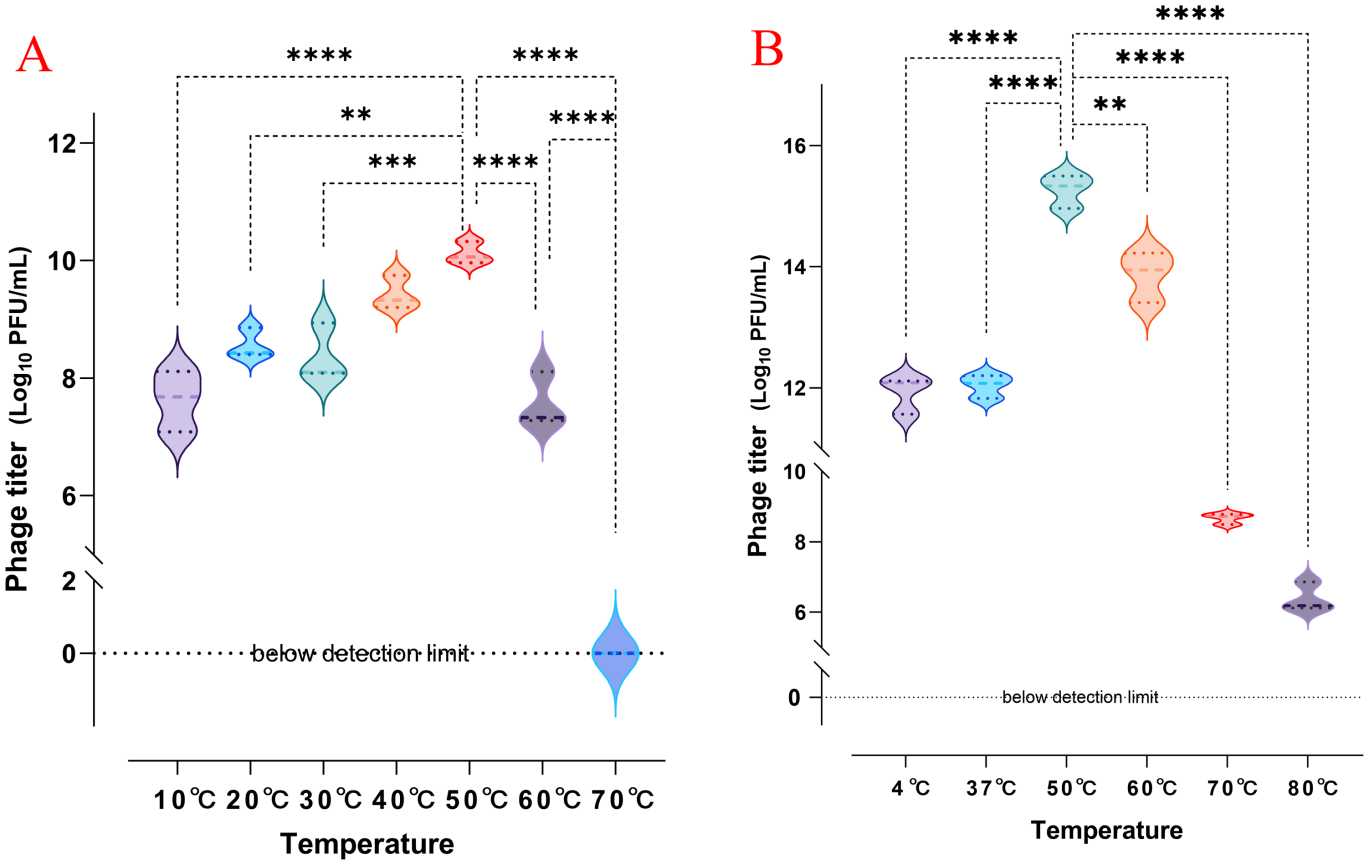
**(A,B)** Changes in phage titer after 1 h of exposure to different temperatures, with initial titers of approximately 10^8^ and 10^12^ PFU/mL, respectively.

Pathogenic *K. pneumoniae* is distributed across various environmental conditions. Therefore, the ability of phages to control these pathogens under different conditions is crucial for their practical application. The tolerance of phage vB_Kp_XP4 at different titers (10^8^ and 10^12^PFU/mL) was tested across a range of temperatures (4°C, 37°C, 40°C, 50°C, 60°C, 70°C, and 80°C). The results showed that phage vB_Kp_XP4 reached its highest titer at 50°C after 1 h. At 70°C for 1 h, phages with a titer of 10^8^ PFU/mL were completely inactivated, while phages with a titer of 10^12^PFU/mL partially survived, and even at 80°C for 1 h, a small amount of phages with this titer still survived (Figure 3).

**Figure 4:**
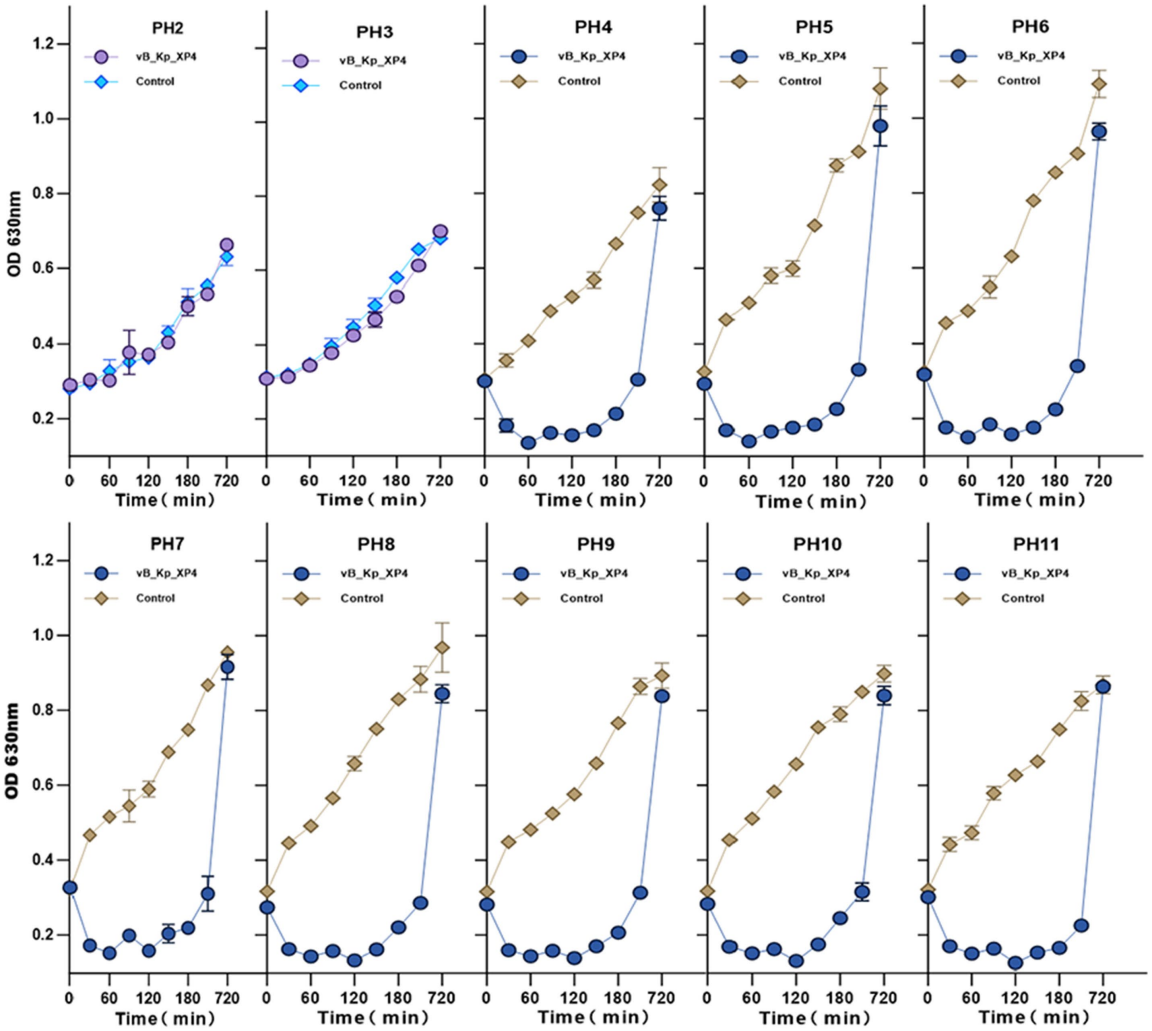
Effect of pH on the ability of phage vB_Kp_XP4 to inhibit the growth of host bacteria. Changes in OD at 630 nm were measured for phage-treated bacterial cultures under different pH conditions, with bacterial cultures without phage treatment used as controls. The experiment was carried out in three technical replicates.

The impact of different pH levels on the phage’s ability to inhibit its host bacteria was analyzed, as shown in Figure 4. The results indicated that at pH ≤ 3, the growth of host bacteria in both the experimental and control groups was significantly inhibited, suggesting that both phage and bacterial growth are restricted by highly acidic conditions. Within a pH range of 4 to 11, the growth of host bacteria in the experimental group (MOI of 0.1) was significantly inhibited, indicating that the phage exhibits antibacterial activity across this broad pH spectrum. Additionally, it was observed that after 12 h, the concentration of host bacteria increased markedly, and the difference between the experimental and control groups diminished. This suggests that the phage was unable to completely eradicate the host bacteria, leading to a relative equilibrium between the two over time.

### 3.7 Phage genome sequencing and characterization

#### 3.7.1 Whole genome basic characteristics

The sequencing results revealed that the genome of phage vB_Kp_XP4 is a linear double-stranded DNA with a genome size of 44,344 bp and a G + C content of 53.80%. A total of 54 coding sequences (CDS) were predicted, all oriented forward (see Supplementary File 1). The online tool tRNAscan-SE predicted that phage vB_Kp_XP4 contains no tRNA genes. Homology analysis using BLAST against the VFDB database found no known virulence genes in phage vB_Kp_XP4. Similarly, analysis with the resfinder database predicted the absence of antibiotic-resistance genes in the genome. The complete nucleotide sequence of phage vB_Kp_XP4 has been submitted to GenBank with the accession number PP663283.

#### 3.7.2 Comparative genomic analysis of phage vB_Kp_XP4

**Figure 5:**
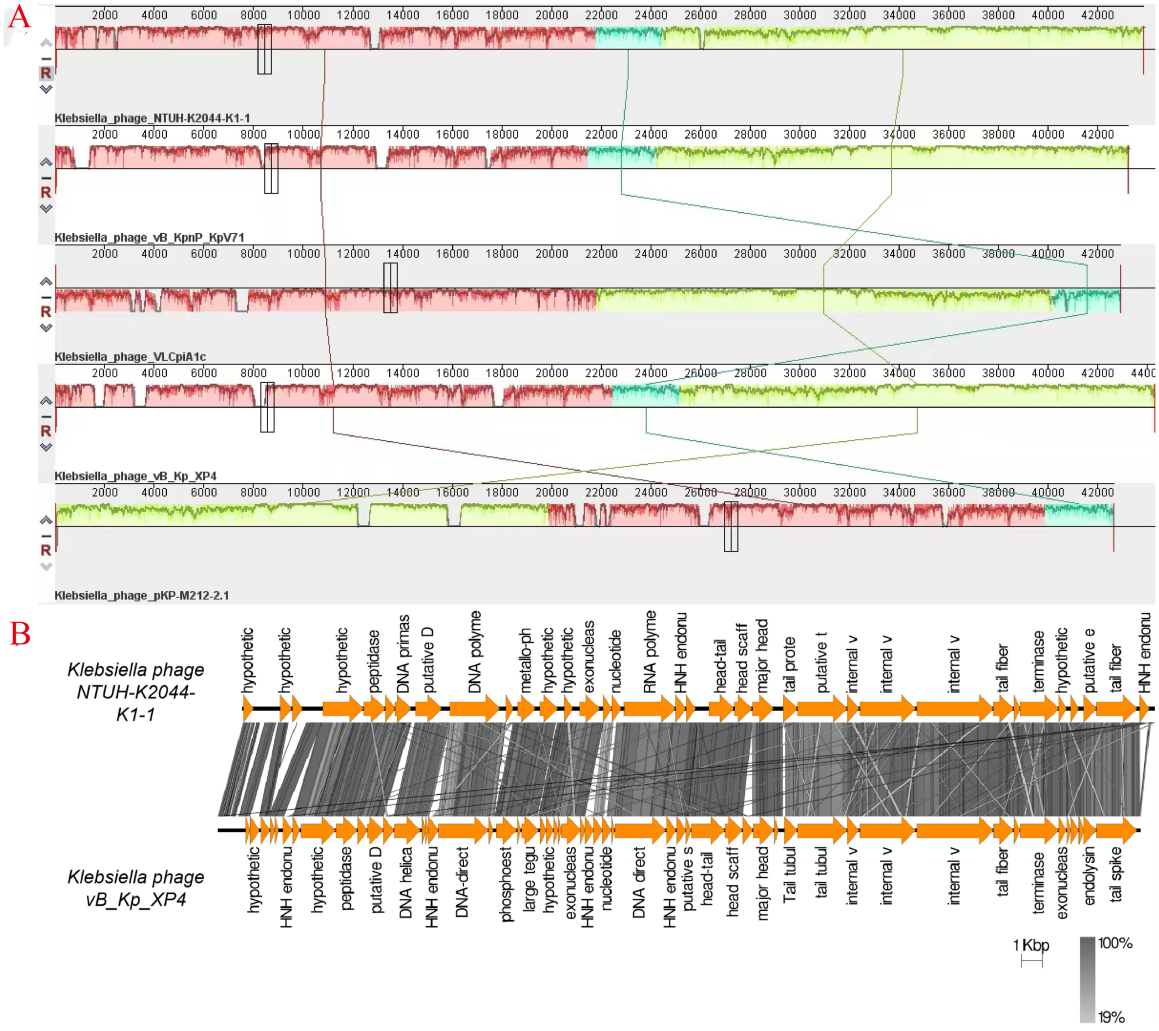
**(A)** Genome comparison of phage vB_Kp_XP4 with phages NTUH-K2044-K1-1, VLCpiA1c, KpV71 and pKP-M212-2.1. Colored blocks indicate regions of similarity between phage genomes, with the height of the panels within the blocks representing the strength of nucleotide similarity; **(B)** Comparison of the phage vB_Kp_XP4 genome with NTUH-K2044-K1-1 using EasyFig software. Arrows indicate predicted CDS based on their genomic functions, while the gray chromatogram reflects genetic similarity percentages.

A whole-genome comparison of phage vB_Kp_XP4 was performed using the BLASTn program on the NCBI website. The results showed a high similarity between phage vB_Kp_XP4 and phages from the genera *Autographiviridae*, *Slopekvirinae*, and *Drulisvirus*. Based on the BLASTn results, the phages with the highest scores, *Klebsiella* phage NTUH-K2044-K1-1, *Klebsiella* phage VLCpiA1c, *Klebsiella* phage KpV71, and *Klebsiella* phage pKP-M212-2.1, were selected for further comparison, as shown in Table 3. Previous studies have shown that (Lin et al., 2014; Solovieva et al., 2018) *Klebsiella* phage NTUH-K2044-K1-1 and KpV71 exhibit lytic activity against the K1 capsular serotype of *K. pneumoniae*. Collinearity analysis using Mauve software (Darling et al., 2004) revealed that certain regions of the *Klebsiella* phage vB_Kp_XP4 genome are similar to those of other *Klebsiella* phage genomes, indicated by the same colors in Figure 5A. The sequences of *Klebsiella* phage vB_Kp_XP4, KpV71, and pKP-M212-2.1 were found to be highly conserved, with no rearrangements, insertions, or inversions, indicating a strong collinearity and similar conserved framework. Additionally, the genome sequence of *Klebsiella* phage vB_Kp_XP4 was compared with that of *Klebsiella* phage NTUH-K2044-K1-1 using EasyFig software (Sullivan et al., 2011) (Figure 5B).

**Table 3:** Whole genome-based databank homologies of *Klebsiella* phage vB_Kp_XP4 according to NCBI.

Strains	Coverage (%)	Identity (%)	Accession number	E
*Klebsiella* phage NTUH-K2044-K1-1	88	90.92	ON602748.1	0
*Klebsiella* phage VLCpiA1c	88	96.39	MK380015.1	0
*Klebsiella* phage KpV71	88	94.97	NC_031246.1	0
*Klebsiella* phage pKP-M212-2.1	89	96.93	OQ734493.1	0

#### 3.7.3 Gene function prediction and annotation

**Figure 6:**
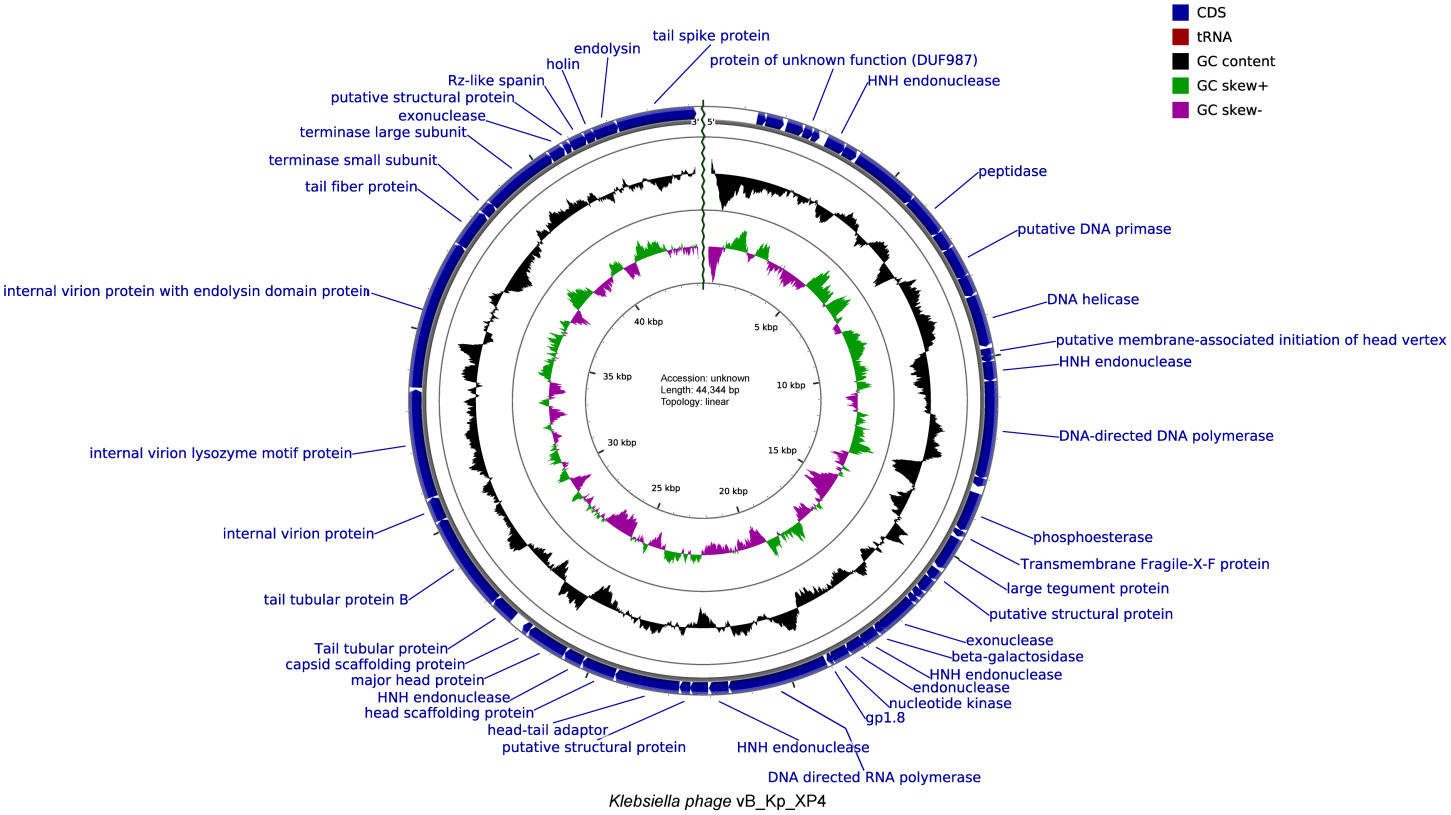
Genomic map of *Klebsiella* phage vB_Kp_XP4 with functional annotation.

**Figure 7:**
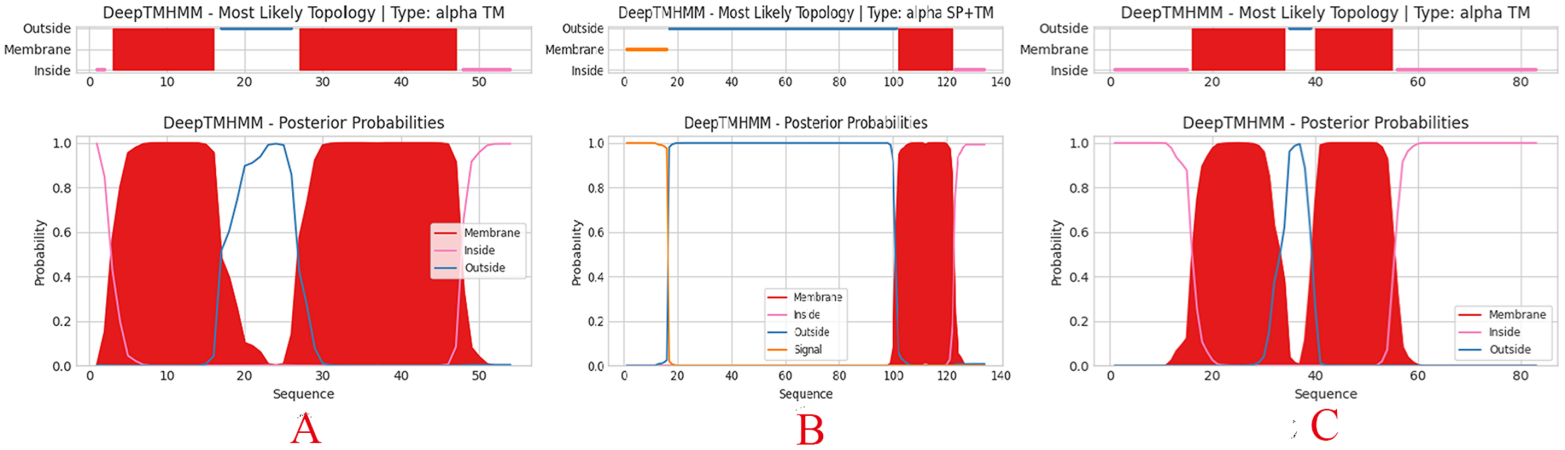
Predicted transmembrane topology of hypothetical holin (CDS20 **(A)**, CDS51 **(B)**, and CDS52 **(C)**) using the DeepTMHMM tool. Red squares represent predicted transmembrane domains, while pink and blue lines represent intra-and extra-membrane domains, respectively. The y-axis represents predicted probability, and the x-axis represents amino acid sequence positions.

The functions of the phage genome were predicted and annotated using Prokka, blastp, and eggNOG-mapper, as shown in Figure 6. Among the 54 analyzed coding sequences (CDS), 49 (90.7%) use ATG as the start codon, three (5.6%)—CDS2, CDS13, and CDS16—use GTG, and two (3.7%)—CDS19 and CDS36—use TTG. Fourteen CDSs were annotated as hypothetical proteins or proteins with unknown functions, while the remaining 40 CDSs have clearly defined functions. These include proteins involved in phage morphology and structure, DNA replication, transcription, packaging, and lysis. Thirteen CDSs are related to capsid and tail structural proteins, while 17 CDSs are associated with replication, transcription, and packaging. Proteins related to phage-mediated lysis of the host include glycosidases, transmembrane proteins, lysozymes, holins, and endolysins. None of the predicted CDSs encode lysogenic phage-associated proteins, such as transposases or integrases. Additionally, a screening of the phage genome with Phage Leads (Yukgehnaish et al., 2022) detected no genes indicative of a temperate lifecycle, antibiotic resistance, or virulence factors. These findings suggest that phage vB_Kp_XP4 has a certain level of safety and applicability for clinical therapeutic use. Various proteins and pathways have been identified as participants in phage-mediated bacterial lysis (Kongari et al., 2018). The holin-endolysin pathway is the most well-known mechanism, with additional involvement from transmembrane proteins. Biochemical and genetic studies indicate that Spanins are essential for disrupting the outer membrane (OM) of Gram-negative hosts. Rz-like proteins, which form two-component transmembrane proteins, are capable of degrading and lysing the OM (Summer et al., 2007). Using DeepTMHMM, the transmembrane domains (TMDs) of predicted proteins were analyzed, revealing three potential proteins (CDS: 20, 51, and 52) with TMDs. CDS51 encodes an Rz-like spanin and was found to have one TMD topology (Figure 7B); CDS20 encodes a transmembrane protein, and CDS52 encodes a holin. Two TMD topologies were detected in the predicted proteins of CDS20 and CDS52 (Figures 7A,C).

#### 3.7.4 Evolutionary analysis of phage vB_Kp_XP4

**Figure 8:**
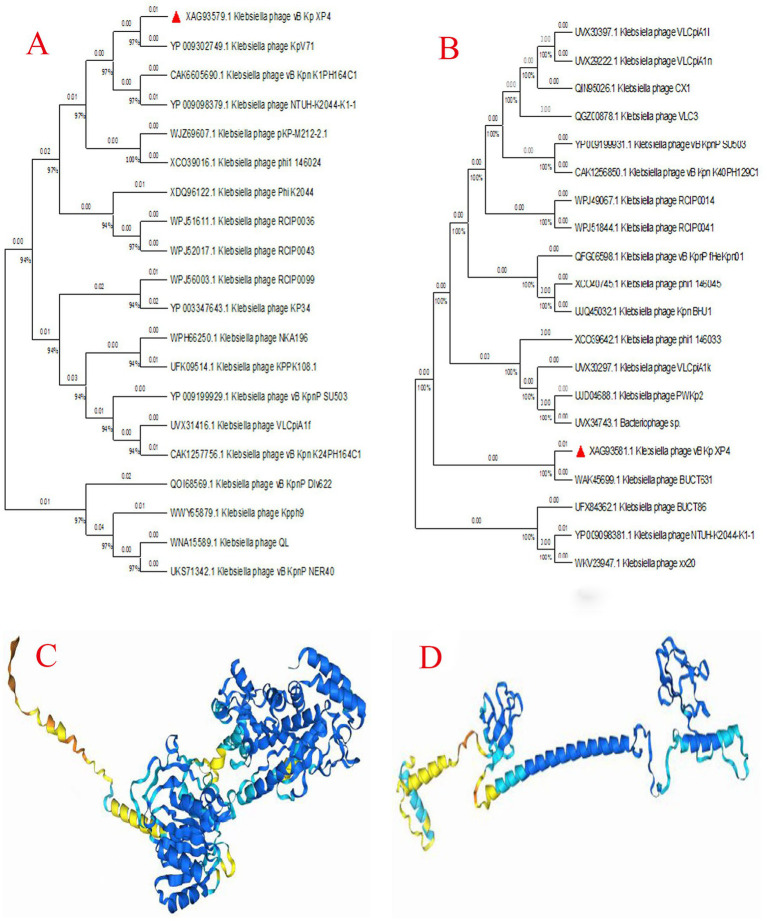
**(A,B)** Phylogenetic tree of the aligned amino acid sequences of key signature proteins, including the terminase large subunit **(A)** and tail fiber protein **(B)**, constructed using the Maximum likelihood method with the JTT matrix-based model and 1,000 replicates in MEGA11; **(C,D)** The predicted protein structures of the terminase large subunit **(C)** and tail fiber protein **(D)**. Deep blue represents “Very high confidence (plDDT>90),” light blue represents “Confidence (90 > plDDT>70),” yellow represents “Low confidence (70 > plDDT>50),” and orange represents “Very low confidence (plDDT<50)”.

To further investigate the evolutionary relationships of phage vB_Kp_XP4, a phylogenetic tree was constructed based on the conserved terminase large subunit (CDS48) using blastp for protein comparison in the NCBI database. The phylogenetic tree (Figure 8A) showed that phage vB_Kp_XP4 is closely related to phage BUCT86 and other phages. To understand the relationship between host specificity and phage tail fiber protein gene sequences, a phylogenetic tree was also constructed using the tail fiber protein (CDS46) (Figure 8B). This analysis revealed that the tail fiber protein sequence had the highest homology with *Klebsiella* phage KpV71, vB_Kpn_K1PH164C1, and NTUH-K2044-K1-1. Overall, phages with high sequence homology belong to the genus *Drulisvirus*, further confirming the close evolutionary relationship of *Klebsiella* phage vB_Kp_XP4 with the *Drulisvirus* genus. Using AlphaFold3, the protein structures of the terminase large subunit and the tail fiber protein of the *Klebsiella* phage vB_Kp_XP4 were predicted (Figures 8C,D).

### 3.8 Assessment of the efficacy of phage vB_Kp_XP4 against strain P4 *in vivo*

**Figure 9:**
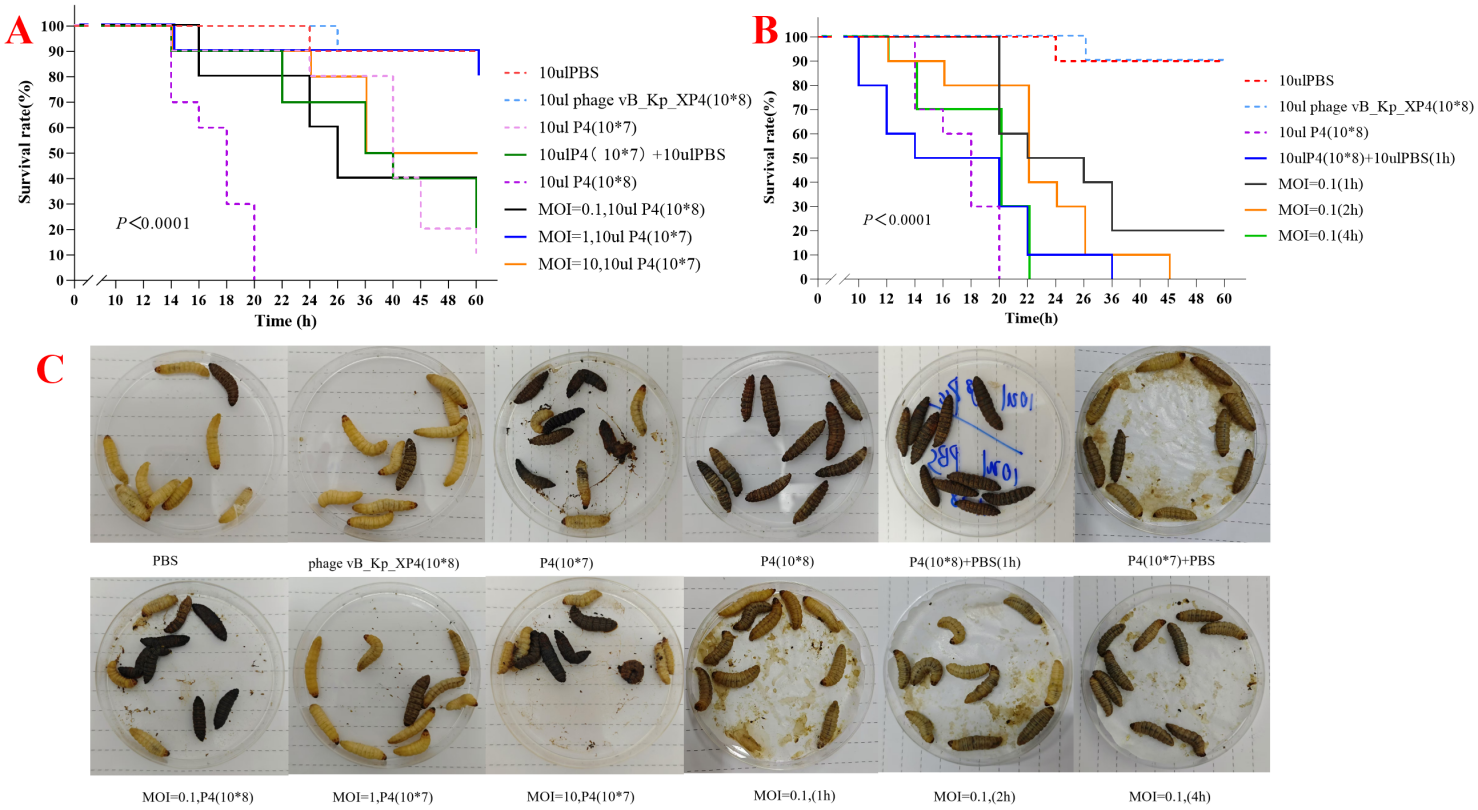
**(A,B)** Survival rates of large wax moth *larvae* treated with varying MOI, time, PBS, and bacteriophages; **(C)** Therapeutic effects of *Klebsiella* phage vB_Kp_XP4 on *larvae* infected with *Klebsiella pneumoniae* P4.

*Galleria mellonella* larvae model was used to assess the efficacy of phage vB_Kp_XP4 against strain P4 *in vivo*. At 60 h, the survival rate of larvae injected with only PBS and phage was both 90%, while the survival rate of larvae injected with strain P4 (10^8^) was 0%, and those injected with strain P4 (10^7^) had a survival rate of 10%. The P4 + PBS group had a survival rate of 20%, while the groups injected with P4 + phage at MOI = 0.1, 1, and 10 had survival rates of 40, 80, and 50%, respectively. Notably, at MOI = 0.1, the lower survival rate was related to the higher concentration of P4 in this group (Figure 9A). It can be observed that the phage treatment groups performed significantly better than the untreated group. Larvae injected with strain P4 (10^8^) all died within 20 h, and those injected with PBS at 1 h post-injection died within 36 h; when MOI = 0.1, larvae injected with phage at 1 h had a survival rate of 20% at 60 h; larvae injected with phage at 2 h all died by 45 h; larvae injected with phage at 4 h all died within 22 h (Figure 9B). It is evident that under the same infection conditions, the earlier phage treatment is administered, the longer the survival time of *Galleria mellonella* larvae, and the more pronounced the therapeutic effect. Additionally, larvae injected with phage alone still had a high survival rate, demonstrating the safety of phage therapy in this model. The morphology of the *Galleria mellonella* larvae after treatment with phage vB_Kp_XP4 is shown in Figure 9C.

## 4 Discussion

It is well known that the phenomenon of “mutual inhibition” among organisms is common. At the end of the 19th century, an outbreak of cholera in the Ganges River mysteriously disappeared, suggesting the presence of a natural antagonist to bacteria in the environment, which was later identified as bacteriophages (d'Herelle, 1931; Duckworth, 1976). In the early 20th century, bacteriophages were widely used to treat various bacterial infections, including cholera, dysentery, plague, Staphylococcus, *Escherichia coli*, and Streptococcus (McCallin et al., 2019; Summers, 1993; Myelnikov, 2020). However, with the advent of antibiotics in the mid-20th century, which marked a “golden era” lasting over 40 years, research on bacteriophages nearly halt (Gordillo Altamirano and Barr, 2019). By the end of the 20th century, the development of new antibiotics had slowed significantly, entering what is often referred to as a “dry pipeline,” while antimicrobial resistance continued to escalate as a global threat (Gordillo Altamirano and Barr, 2019; Hitchcock et al., 2023). With rapid advancements in biology, medicine, and other fields, our understanding and research on bacteriophages have deepened, and phage therapy has re-emerged as a promising solution to mitigate the antibiotic resistance crisis.

In this study, a lytic environmental phage was isolated from a natural water source using the hypervirulent *K. pneumoniae* strain P4 as the host. At 36°C, within 12 h, the phage formed plaques approximately 2 mm in diameter with surrounding halos consisting of multiple semi-transparent layers. As time progressed, the halos expanded, and by 36 h, the plaques and halos reached a diameter of up to 28 mm, consistent with the findings of Zaki et al. (2023). According to morphological observations and the latest classification data from the International Committee on Taxonomy of Viruses (ICTV),^4^ most phages isolated from *K. pneumoniae* are double-stranded DNA phages belonging to the order Caudovirales (Zerbini et al., 2023).

The lysis efficiency of phage vB_Kp_XP4 is positively correlated with temperature between 4°C and 50°C. However, when the temperature exceeds 60°C, this relationship reverses, regardless of whether the titer is high (10^12^ PFU/ml) or low (10^8^ PFU/ml). It is similar to the behavior observed in phage vB_KpnP_IME337 (Gao et al., 2020). Increasing the initial dose of the phage allows some activity to be retained, suggesting that adjusting the phage dosage could effectively target heat-resistant bacterial strains in high-temperature environments. The phage also demonstrated strong lytic activity across a pH range of 4 to 11, indicating high stability under various conditions. This stability suggests that Phage vB_Kp_XP4 could be useful not only under standard conditions but also in special environments, such as in the disinfection and cleaning of hospital settings contaminated with multidrug-resistant bacteria (Otter et al., 2015) or in applications within high-temperature, acidic, or alkaline environments (Suja and Gummadi, 2023). These properties confer broad practicality and application value to the phage. The study found that phage vB_Kp_XP4 has a latent period of approximately 10 min, shorter than most phages (Li et al., 2020), allowing for quicker control of pathogenic bacterial infections. The phage titer peaked around 50 min, entering a plateau phase. Due to the strong viscosity of the host bacteria, some free phages were not fully eluted during the experiment, slightly affecting the measurement of the burst size. However, the rapid and efficient lytic capability of the phage remains evident. Lytic phages with short incubation times, meaning they have a rapid replication cycle within the host bacterium, and high productivity, indicating they produce a large number of new phage particles per infected host cell, such as vB_Kp_XP4, are suitable for use during the acute phase of infections (Meile et al., 2022), effectively reducing the number of pathogens and providing more time for the host immune response and clinical treatments, thereby improving patient outcomes. After 100 min, a marked decline in phage titer and an increase in bacterial count were observed, which aligns with the growth pattern of phage ST11 K47 (Fang and Zong, 2022). Continued observation in liquid culture showed that the numbers of phages and bacteria tended to stabilize, indicating that in liquid media, phages cannot eliminate bacteria, as the death of bacteria would also disrupt the phage’s limited food chain. On solid plates, when the phage titer reaches a sufficient level, bacterial growth can be completely inhibited, demonstrating that the growth and mutation rates of bacteria and phages are significantly influenced by the culture environment (Chevallereau et al., 2022). On this basis, we adopted *Galleria mellonella* larvae as the infection model. The natural immune system of *Galleria mellonella* has similarities with the human immune system and is easy to obtain and breed (Feng et al., 2023). In this study, a new phage vB_Kp_XP4 was used to prolong the survival time of *Galleria mellonella* larvae after infection. The survival time of the phage treatment group was significantly longer than that of the non-intervention group, and the earlier the phage intervention was carried out, the more obvious the treatment effect was, which was consistent with the conclusion of a certain study (Lin et al., 2014). Further *in vivo* studies are needed to explore the interaction mechanisms between phages and bacteria. Regarding the emergence of phage-resistant bacterial strains, many studies have shown that the acquisition of phage resistance often results in a significant decrease in virulence and antibiotic resistance (Chen et al., 2024). Combining antibiotics (Ziller et al., 2024) and phage cocktails (Yoo et al., 2024) can suppress the emergence of resistant strains. A substantial body of animal studies has demonstrated that the combination of phages, antibiotics, and the host immune system can effectively control bacterial infections (Tang et al., 2024; Pal et al., 2024; Nang et al., 2024). However, clinical cases are relatively limited, and more clinical trials are necessary to optimize, validate, and refine the therapeutic use of phages. *Klebsiella* phage vB_Kp_XP4 shows a narrow lytic spectrum and high specificity, typical of natural phages (Dion et al., 2020). Phages rely primarily on the specificity of their tail structures to recognize host bacteria. Modifying phages through induced culture, co-culture, and genetic engineering strategies can help broaden their lytic spectra (Parker and Nugen, 2024; Ulrich et al., 2024; Hesse et al., 2020).

Based on whole-genome sequencing data, a comparison using Blastn in the NCBI database revealed that *Klebsiella* phage vB_Kp_XP4 is most similar to *Klebsiella* phage NTUH-K2044-K1-1 (ON602748.1), with 88% coverage, 90.92% identity, and an E-value of 0. The nucleotide sequence of *Klebsiella* phage vB_Kp_XP4 differs by more than 5% from that of known phages, indicating it represents a new species (Grigson et al., 2023). Further phylogenetic analysis using conserved proteins confirmed that *Klebsiella* phage vB_Kp_XP4 is closely related to phages in the genus Drulisvirus. Therefore, it is suggested that *Klebsiella* phage vB_Kp_XP4 is a new member of the genus Drulisvirus within the subfamily Slopekvirinae. *Klebsiella* phage vB_Kp_XP4 has relatively small nucleic acid molecular weights, making them easier to edit. This characteristic makes them ideal model phages for genetic engineering and synthetic biology applications, offering significant potential for more in-depth research (Lenneman et al., 2021).

The formation of halos around plaques is likely related to the synthesis of phage protein products, including lytic enzymes, endolysins, and spanins (holin, endolysin, and spanin). Numerous studies have demonstrated that phage depolymerases possess biofilm-degrading properties (Tagliaferri et al., 2019; Gliźniewicz et al., 2023; Drulis-Kawa et al., 2015). According to the National Institutes of Health, more than 80% of bacterial diseases are associated with biofilms (Evans et al., 2023). The significant morphological changes observed in the P4 strain after treatment with phage vB_Kp_XP4 suggest its ability to remove the capsule of serotype K1 *Klebsiella pneumoniae*. Phage vB_Kp_XP4 encodes endolysin (CDS53), a cell wall hydrolase synthesized in the late stage of phage infection that hydrolyzes peptidoglycan to release progeny phages (Cahill and Young, 2019; Marques et al., 2021). Euler et al. (2023) demonstrated in mouse experiments that phage endolysins have bactericidal effects against multiple Gram-negative ESKAPE pathogens. Chen et al. (2024) ngineered endolysins with different protein peptides, showing significant bactericidal activity against ESKAPEE pathogens. Endolysins in *Klebsiella* phages exhibit diversity and conservation within the genus, providing substantial potential for further exploration (Chang et al., 2022). Future research will focus on phage protein products, such as depolymerases and endolysins, to develop formulations targeting bacterial biofilms and novel antibacterial agents. Such developments could provide a material basis for combining phages and antibiotics, offering more options for treating bacterial infections. In this study, we observed the morphological differences of the strains before and after phage treatment using only light microscopy. Utilizing electron microscopy may provide more detailed insights. As a biological therapy, phage treatment must prioritize safety considerations. Comparisons with existing databases indicate that *Klebsiella* phage vB_Kp_XP4 does not contain tRNA, lysogenic genes, antibiotic resistance genes, or virulence genes, thereby posing no risk of transmitting resistance or virulence genes, which ensures its safety for clinical applications.

### 5 Conclusion

In conclusion, bacteriophages and their protein products have garnered significant interest globally as potential therapies to reduce or replace antibiotic use. This study successfully isolated a novel *Klebsiella* phage vB_Kp_XP4, and characterized its biological properties and genomic features. *In vivo* experiments demonstrated its therapeutic effect on a *Galleria mellonella* infection model, with high safety and efficacy, making it a promising candidate for phage therapy and a potential synergist in combination with antibiotics, offering additional options for antimicrobial treatment. Furthermore, phage protein products, such as lytic enzymes and endolysins, possess properties that assist in bacterial lysis. Future *in vitro* and *in vivo* experiments could explore the antibacterial effects of phage suspensions, protein synthesis products, and various combinations with antibiotics.

## Data Availability

The datasets presented in this study can be found in online repositories. The names of the repository/repositories and accession number(s) can be found in the article/Supplementary material.

## Footnotes

^1^ http://lowelab.ucsc.edu/tRNAscan-SE/

^2^ https://services.healthtech.dtu.dk/services/DeepTMHMM-1.0/

^3^ http://www.ncbi.nlm.nih.gov/BLAST

^4^ http://ictv.global/taxonomy
